# Functional recovery patterns from discharge to 12 months after endovascular treatment for aneurysmal subarachnoid hemorrhage: a multicenter cohort study

**DOI:** 10.3389/fnins.2026.1935474

**Published:** 2026-09-03

**Authors:** Qiao Wu, LongXiang Ma, Yang Chen, ZhenRan Zhao, JinFang Liu, Bin Zhang, HeChun Xia

**Affiliations:** 1 Department of Neurosurgery, Ningxia Medical University General Hospital, Yinchuan, China; 2 Ningxia Key Laboratory of Stem Cell and Regenerative Medicine, Institute of Medical Sciences, General Hospital of Ningxia Medical University, Yinchuan, China; 3 Department of Neurology, General Hospital of Ningxia Medical University, Yinchuan, China; 4 Department of Neurointervention, General Hospital of Ningxia Medical University, Yinchuan, China

**Keywords:** aneurysmal subarachnoid hemorrhage, endovascular treatment, functional recovery pattern, modified Rankin Scale, persistent functional disability

## Abstract

**Background:**

Functional status after aneurysmal subarachnoid hemorrhage (aSAH) may improve after discharge, but paired assessments at discharge and long-term follow-up have rarely been used to distinguish delayed recovery from persistent disability. We characterized discharge-to-12-month recovery patterns after endovascular treatment and identified factors associated with persistent disability among patients with poor functional status at discharge.

**Methods:**

This multicenter retrospective cohort study consecutively enrolled patients with aSAH who underwent endovascular treatment between April 2021 and April 2024 and had available mRS data at discharge and 12-month follow-up. Demographic characteristics, medical history, aneurysm-related features, laboratory parameters, clinical severity, and perioperative data were collected. Based on mRS scores at discharge and 12 months, patients were classified as persistently favorable, delayed recovery, or persistently poor. Among patients with poor functional status at discharge, univariable and multivariable logistic regression analyses were performed to identify factors associated with persistent functional disability.

**Results:**

Among 1,637 patients, 1,328 (81.1%) were persistently favorable. Of 309 patients with poor functional status at discharge, 116 (37.5%) recovered functional independence by 12 months and 193 (62.5%) remained disabled. In the fully adjusted model, monocyte count (OR = 1.02, 95% CI: 1.00–1.04), modified World Federation of Neurosurgical Societies (mWFNS) grade (OR = 1.67, 95% CI: 1.29–2.18), and external ventricular drainage (EVD; OR = 3.71, 95% CI: 1.47–10.49) were associated with higher odds of persistent disability, whereas the systemic inflammation response index was associated with lower odds (OR = 0.99, 95% CI: 0.98–1.00).

**Conclusion:**

Substantial heterogeneity exists in post-discharge functional recovery after endovascular treatment for aSAH. Clinical severity, external ventricular drainage, and inflammation-related indicators were associated with persistent disability. Recovery patterns based on mRS trajectories may support post-discharge risk stratification and rehabilitation follow-up.

## 1 Introduction

Aneurysmal subarachnoid hemorrhage (aSAH) is a cerebrovascular emergency associated with high mortality and substantial disability (Hoh et al., 2023). With advances in microsurgical techniques, endovascular therapy, and neurocritical care, an increasing number of patients survive the acute phase and enter a prolonged period of functional recovery. However, successful aneurysm occlusion does not necessarily indicate that functional outcome has already been determined (Hammer et al., 2020). Previous studies have shown that modified Rankin Scale (mRS) scores may continue to improve after discharge in patients with aSAH, and functional recovery in some patients may extend for several years after onset (Lee et al., 2025; Wilson et al., 2013). Therefore, functional outcome after aSAH should be viewed as a dynamic recovery process rather than a static status assessed at a single time point.

Previous studies have evaluated functional outcomes after aSAH at predefined time points, including discharge and 3-, 6-, or 12-month follow-up, and have identified multiple factors associated with prognosis (Nobels-Janssen et al., 2024). These studies have provided important evidence for prognostic assessment; however, an important clinical question remains: among patients who are already functionally dependent at discharge, which patients subsequently regain functional independence and which remain persistently disabled? Few studies have specifically focused on post-discharge transitions in functional status within this subgroup. Linking discharge status with subsequent long-term outcome may therefore complement conventional fixed-time-point prognostic assessment by providing additional information on recovery potential among patients with an initially poor functional status at discharge.

Among patients with poor functional status at discharge, subsequent recovery may be associated with several clinical domains. Clinical severity at admission, assessed using the modified World Federation of Neurosurgical Societies (mWFNS) grade, reflects the burden of early brain injury and systemic stress (Sano et al., 2015). Acute hydrocephalus and the need for external ventricular drainage may indicate impaired cerebrospinal fluid circulation and a more complicated neurocritical-care course (Nelson et al., 2022). Inflammation- and nutrition-related biomarkers may also be associated with secondary brain injury and tissue repair processes (Dhar and Diringer, 2008; Luo et al., 2025; Yun et al., 2021). However, evidence remains limited regarding the factors that distinguish subsequent recovery from persistent disability among patients who are already functionally dependent at discharge.

Accordingly, using data from a multicenter cohort, we combined discharge and 12-month mRS assessments to characterize three transition-based functional recovery patterns: persistently favorable, delayed recovery, and persistently poor. More importantly, the primary analysis focused on patients with poor functional status at discharge (mRS 3–6) to identify factors associated with persistent disability versus subsequent recovery at 12 months. By shifting the focus from a single long-term outcome in the overall cohort to recovery potential among patients with an initially poor discharge status, this study aimed to provide complementary evidence for post-discharge prognostic counseling, follow-up planning, and rehabilitation management after aSAH.

## 2 Materials and methods

### 2.1 Study design and population

This multicenter retrospective cohort study was conducted at five tertiary medical centers in China and consecutively enrolled patients with aSAH who underwent endovascular treatment between April 1, 2021, and April 30, 2024. The diagnosis of aSAH was established on the basis of compatible clinical manifestations and evidence of subarachnoid hemorrhage on cranial computed tomography (CT) or other neuroimaging examinations, together with confirmation of a causative intracranial aneurysm by computed tomography angiography (CTA), magnetic resonance angiography (MRA), or digital subtraction angiography (DSA) (Westerlaan et al., 2011). Endovascular treatment modalities included primary coil embolization, stent-assisted coil embolization, and flow-diverter implantation (Adamou et al., 2021).

**FIGURE 1:**
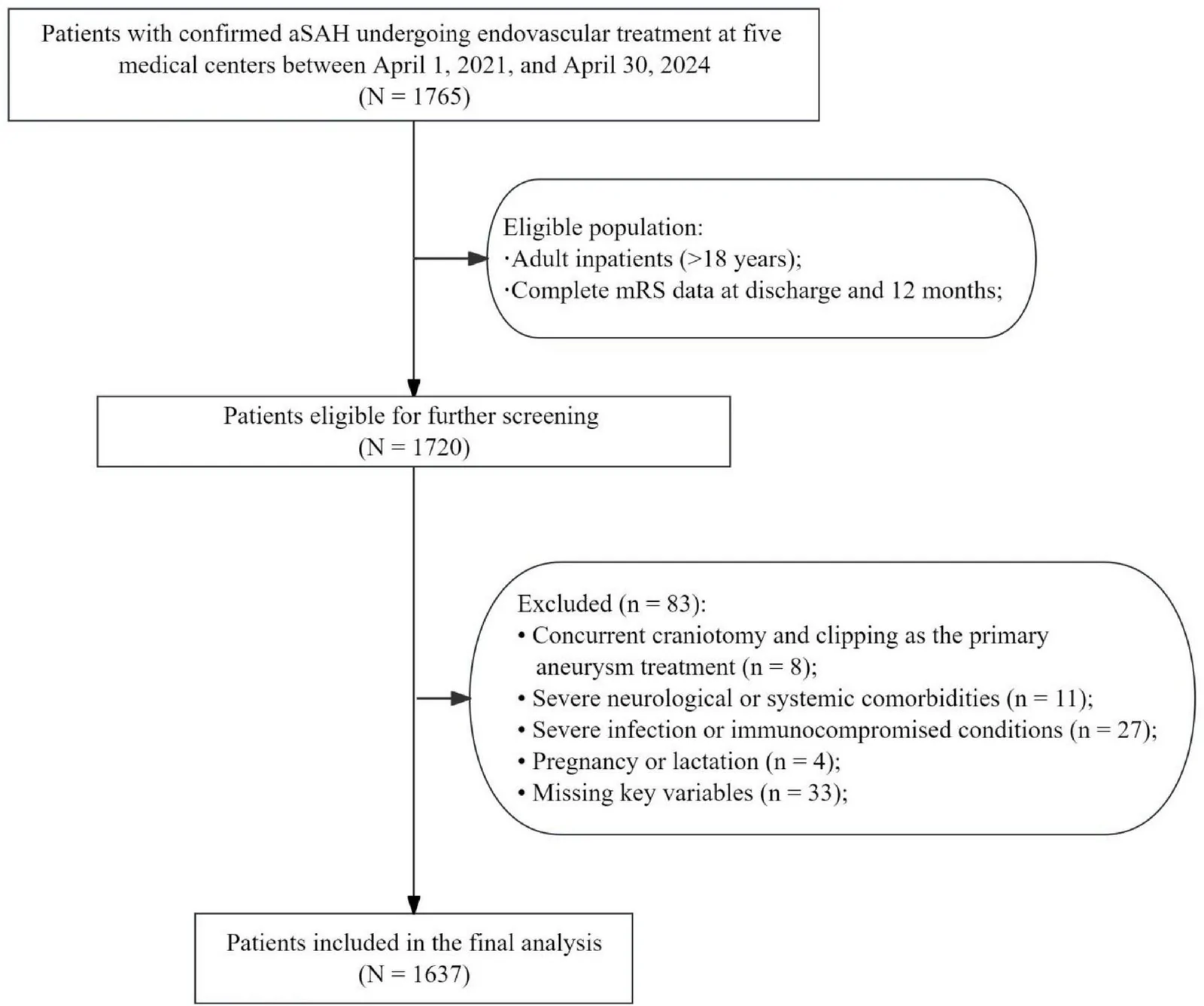
Flowchart of patient selection. The five centers were the Second Affiliated Hospital of Nanchang University, the First Affiliated Hospital of Nanchang University, Henan Provincial People’s Hospital, the First Affiliated Hospital of Nanyang Medical College, and the First Affiliated Hospital of Zhengzhou University.

The inclusion criteria were as follows: (1) age > 18 years; (2) a confirmed diagnosis of aSAH; (3) receipt of endovascular treatment during the index hospitalization; and (4) complete mRS data at discharge and at the 12-month follow-up. The exclusion criteria were: (1) nonaneurysmal subarachnoid hemorrhage; (2) microsurgical clipping performed as the primary treatment for the causative aneurysm during the index hospitalization; (3) severe neurological or systemic comorbidities that could substantially interfere with the assessment of functional outcomes; (4) severe infection or an immunocompromised condition; (5) pregnancy or lactation; and (6) missing key variables that precluded classification of functional recovery patterns or completion of the primary statistical analyses. After applying these eligibility criteria, 1,637 patients were included in the overall cohort analysis. The patient selection process is presented in Figure 1.

### 2.2 Data collection

Baseline characteristics, aneurysm-related features, preoperative laboratory parameters, clinical severity, treatment information, and perioperative data were collected from the electronic medical records, imaging databases, and follow-up records of the participating centers.

Demographic variables included age and sex. Medical history included prior cerebral hemorrhage, cerebral ischemia, heart disease, hypertension, hyperlipidemia, and diabetes mellitus, while lifestyle-related variables included smoking and alcohol consumption. Aneurysm-related characteristics comprised maximum aneurysm diameter, neck width, dome-to-neck ratio, parent artery diameter, aneurysm morphology, bifurcation location, and anatomical site. Aneurysm morphology was categorized as saccular or fusiform. Anatomical locations were classified as the anterior communicating artery–anterior cerebral artery complex, internal carotid artery–posterior communicating artery segment, anterior cerebral artery, middle cerebral artery, basilar artery, or other locations.

Preoperative laboratory parameters included neutrophil, lymphocyte, monocyte, and platelet counts; C-reactive protein (CRP); albumin; high-density lipoprotein cholesterol (HDL-C); fibrinogen; and D-dimer. The following composite indices were subsequently calculated: fibrinogen-to-HDL-C ratio (FHR) (Jia et al., 2024), monocyte-to-lymphocyte ratio (MLR), neutrophil-plus-monocyte-to-lymphocyte ratio (nMLR) (Feghali et al., 2021), platelet-to-lymphocyte ratio (PLR) (Tao et al., 2017), neutrophil-to-albumin ratio (NAR) (Zhang et al., 2023), neutrophil-to-lymphocyte ratio (NLR) (Cai et al., 2021), CRP-to-albumin ratio (CAR) (Zhang et al., 2019), monocyte-to-HDL-C ratio (MHR) (Liu et al., 2020), systemic immune-inflammation index (SII; neutrophil count × platelet count/lymphocyte count) (Luo et al., 2022), systemic inflammation response index (SIRI; neutrophil count × monocyte count/lymphocyte count) (Yi et al., 2024), D-dimer-to-albumin ratio (DAR) (Wu et al., 2022), and prognostic nutritional index (PNI; albumin + 5 × lymphocyte count) (Fan et al., 2023).

Clinical severity was assessed using the mWFNS grade (Nguyen et al., 2023), whereas the radiographic severity of hemorrhage was evaluated using the modified Fisher grade (Frontera et al., 2006). Perioperative variables included the endovascular treatment modality, postoperative complications, perioperative neurosurgical procedures, length of stay in the intensive care unit (ICU), time from symptom onset to intervention, procedure duration, immediate postprocedural Raymond–Roy occlusion grade, and intraoperative antiplatelet or antithrombotic therapy. Perioperative neurosurgical procedures included lumbar puncture, lumbar drainage, external ventricular drainage (EVD), ventriculoperitoneal shunting, decompressive craniectomy, or no additional neurosurgical procedure (Diringer et al., 2011; Treggiari et al., 2023). Intraoperative antiplatelet or antithrombotic therapy primarily referred to tirofiban infusion administered during the procedure (Yoon et al., 2018). Postoperative complications included, but were not limited to, pneumonia, peptic ulcer disease, congestive heart failure, aneurysmal rebleeding, anemia, seizures, vasospasm, glomerulonephritis, postoperative cerebral infarction, intracranial infection, and hydrocephalus. For each patient, we calculated the total number of postoperative complications, which ranged from 0 to 9. Patients who experienced ≥ 2 complications (approximately corresponding to the ≥ 75th percentile of the distribution) were classified as the high-complication group, whereas those with 0–1 complication were classified as the low-complication group (Sonig et al., 2018).

### 2.3 Functional outcome and imaging assessment

The discharge mRS score was assigned by a board-certified neurosurgeon based on the neurological examination and assessment of activities of daily living at discharge. The 12-month mRS score was obtained through a structured telephone interview conducted by trained follow-up physicians who had received standardized training in mRS assessment. The presence, location, morphology, and relevant imaging characteristics of the culprit aneurysm were independently reviewed on CTA, MRA, or DSA by two neurointerventionalists, each with 5 years of experience. Any disagreements were resolved by consensus.

### 2.4 Definition of functional recovery patterns

Functional outcomes were assessed using the mRS. An mRS score of 0–2 was defined as a favorable outcome or functional independence, whereas an mRS score of 3–6 was defined as a poor outcome or functional disability (Banks and Marotta, 2007). According to changes in mRS scores between discharge and the 12-month follow-up, patients in the overall cohort were classified into three *a priori* transition-based functional recovery patterns: persistently favorable, delayed recovery, and persistently poor.

The persistently favorable pattern was defined as an mRS score of 0–2 at discharge and 0–2 at 12 months, indicating that patients had achieved functional independence by discharge and maintained favorable functional status during follow-up. The delayed recovery pattern was defined as an mRS score of 3–6 at discharge that improved to 0–2 at 12 months, indicating that patients had functional disability at discharge but regained functional independence during follow-up. The persistently poor pattern was defined as an mRS score of 3–6 at both discharge and 12 months, indicating persistent functional disability throughout the post-discharge period.

### 2.5 Statistical analysis

Missingness was first evaluated for all variables. Variables with a missing rate of < 20% were imputed using a least-squares-based imputation method, whereas variables with a missing rate of ≥ 20% were excluded from subsequent statistical analyses (Donders et al., 2006). The normality of continuous variables was evaluated by the Shapiro–Wilk test and Q–Q plots. Because the continuous variables were non-normally distributed, they were presented as medians with interquartile ranges [M (IQR)]. Categorical variables were summarized as counts and percentages [n (%)] (Vickers, 2005). Comparisons of continuous variables among the three functional recovery patterns were performed using the Kruskal–Wallis test, whereas categorical variables were compared using the χ^2^ test or Fisher’s exact test, as appropriate (Kim, 2017).

To further identify factors associated with persistent functional disability at 12 months among patients who had poor functional status at discharge, and to distinguish patients with subsequent recovery potential from those at higher risk of unfavorable long-term outcomes, the primary regression analysis was restricted to patients with poor functional status at discharge, namely the delayed recovery and persistently poor groups. Logistic regression analysis was performed to evaluate factors associated with persistent functional disability, with the delayed recovery group as the reference outcome and the persistently poor group as the event outcome.

Univariable logistic regression analysis was first performed to examine the association between each variable and persistent functional disability. Variables with *P*-values < 0.05 in the univariable analysis were considered candidate variables for multivariable analysis. To minimize the influence of multicollinearity on the stability of the multivariable model, Spearman correlation analysis and variance inflation factor (VIF) assessment were conducted for the candidate variables. A VIF > 10 was considered indicative of severe multicollinearity, and the corresponding variables were excluded from the multivariable logistic regression model (O’brien, 2007).

Candidate variables without severe multicollinearity were subsequently included in the multivariable logistic regression models. Three models were constructed: the crude model included only the candidate variables; Model 2 was further adjusted for sex; and the fully adjusted model was additionally adjusted for medical history, including prior cerebral hemorrhage, cerebral ischemia, heart disease, hypertension, hyperlipidemia, and diabetes mellitus. Regression results were reported as odds ratios (ORs) with 95% confidence intervals (CIs). All statistical tests were two-sided, and *P-*values < 0.05 were considered statistically significant. All statistical analyses were performed using R software (version4.4.3).

## 3 Results

### 3.1 Overall clinical characteristics of the study population

A total of 1,637 patients with aSAH who underwent endovascular treatment were included in the final analysis. The overall clinical characteristics of the study population are presented in Table 1. The median age of the cohort was 58.00 (51.00, 66.00) years, and 1,069 patients (65.3%) were female. Hypertension was the most common medical history, accounting for 49.7% of the cohort. The median maximum aneurysm diameter was 5.00 (3.70, 6.82) mm, and saccular aneurysms accounted for 72.4% of cases. The most frequent aneurysm location was the anterior communicating artery–anterior cerebral artery complex, observed in 45.9% of patients. The median preoperative C-reactive protein and albumin levels were 12.40 (4.08, 25.70) mg/L and 39.80 (36.70, 42.85) g/L, respectively. Regarding clinical severity, 73.1% of patients were classified as mWFNS grades I–II, and 67.6% were classified as modified Fisher grades I–II. The predominant endovascular treatment modalities were stent-assisted embolization (52.1%) and primary coil embolization (47.3%). Patients with severe postoperative complications accounted for 6.0% of the cohort, and immediate postprocedural Raymond–Roy grade I occlusion was achieved in 86.8% of patients.

**TABLE 1:** Characteristics of the overall cohort.

Variable	Overall (*N* = 1637)
Age (years), median (IQR)	58.00 (51.00, 66.00)
Sex, *n* (%)	
Male	568 (34.7%)
Female	1069 (65.3%)
Medical history, n (%)	
Cerebral hemorrhage	52 (3.2%)
Cerebral ischemia	85 (5.2%)
Heart disease	75 (4.6%)
Hypertension	813 (49.7%)
Hyperlipidemia	78 (4.8%)
Diabetes	116 (7.1%)
Smoking, *n* (%)	631 (38.5%)
Drinking alcohol, *n* (%)	575 (35.1%)
Aneurysm characteristics	
Aneurysm diameter (mm), median (IQR)	5.00 (3.70, 6.82)
Neck width (mm), median (IQR)	3.15 (2.43, 4.23)
Dome-to-neck ratio, median (IQR)	1.41 (1.07, 1.88)
Parent artery diameter (mm), median (IQR)	2.65 (2.10, 3.26)
Aneurysm morphology, n (%)	
Saccular aneurysm	1186 (72.4%)
Fusiform aneurysm	451 (27.6%)
Bifurcation status, n (%)	
Located at an arterial bifurcation	660 (40.3%)
Not located at an arterial bifurcation	977 (59.7%)
Aneurysm location, n (%)	
ACoA–ACA	751 (45.9%)
PCoA	34 (2.1%)
ACA	54 (3.3%)
ICA	286 (17.5%)
MCA	154 (9.4%)
BA	59 (3.6%)
Other	299 (18.3%)
Preoperative laboratory parameters	
Neutrophils ( × 10^∧^9/L), median (IQR)	8.07 (5.91, 11.04)
Lymphocytes ( × 10^∧^9/L), median (IQR)	1.06 (0.73, 1.46)
Monocytes ( × 10^∧^9/L), median (IQR)	0.60 (0.38, 2.14)
Platelets ( × 10^∧^9/L), median (IQR)	189.00 (107.00, 249.00)
C-reactive protein (mg/L), median (IQR)	12.40 (4.08, 25.70)
Albumin (g/L), median (IQR)	39.80 (36.70, 42.85)
HDL-C (mmol/L), median (IQR)	1.28 (1.16, 1.41)
Fibrinogen (g/L), median (IQR)	3.02 (2.53, 3.55)
D-dimer (mg/L), median (IQR)	1.33 (0.63, 3.02)
FHR, median (IQR)	2.35 (1.92, 2.94)
MLR, median (IQR)	0.55 (0.32, 2.56)
nMLR, median (IQR)	10.70 (5.24, 35.57)
PLR, median (IQR)	157.89 (68.39, 246.46)
NAR, median (IQR)	0.208 (0.149, 0.280)
NLR, median (IQR)	7.70 (4.42, 13.28)
CAR, median (IQR)	0.303 (0.102, 0.658)
MHR, median (IQR)	0.490 (0.292, 1.951)
SII, median (IQR)	1076.70 (274.75, 2202.00)
SIRI, median (IQR)	4.93 (2.15, 26.13)
DAR, median (IQR)	0.033 (0.015, 0.078)
PNI, median (IQR)	45.50 (42.00, 48.90)
mWFNS grade, n (%)	
I	379 (23.2%)
II	817 (49.9%)
III	218 (13.3%)
IV	166 (10.1%)
V	57 (3.5%)
Modified Fisher grade, n (%)	
I	218 (13.3%)
II	889 (54.3%)
III	280 (17.1%)
IV	250 (15.3%)
Intervention approach, n (%)	
Coil embolization	774 (47.3%)
Stent-assisted embolization	853 (52.1%)
Flow-diverter treatment	10 (0.6%)
Postoperative complications, n (%)	
Low complication group	1538 (94.0%)
High complication group	99 (6.0%)
Perioperative surgical operation, n (%)	
Lumbar puncture	306 (18.7%)
Lumbar drainage	63 (3.8%)
External ventricular drainage	61 (3.7%)
Ventriculoperitoneal shunt	7 (0.4%)
Craniotomy (decompressive craniectomy)	23 (1.4%)
No additional neurosurgical procedure	1177 (71.9%)
ICU length of stay (days), median (IQR)	1.00 (1.00, 2.00)
Onset-to-surgery time (days), n (%)	
≤ 1	517 (31.6%)
2–3	390 (23.8%)
4–7	340 (20.8%)
> 7	390 (23.8%)
Operative duration (hours), n (%)	
≤ 1	514 (31.4%)
2–3	532 (32.5%)
4–5	190 (11.6%)
5–6	56 (3.4%)
6–7	295 (18.0%)
> 7	50 (3.1%)
Treatment status, n (%)	
Raymond–Roy I	1421 (86.8%)
Raymond–Roy II	183 (11.2%)
Raymond–Roy III	33 (2.0%)
Antiplatelet or antithrombotic therapy, n (%)	
Yes	484 (29.6%)
No	1153 (70.4%)

Data are presented as median [interquartile range (IQR)] for continuous variables and *n* (%) for categorical variables. Percentages were calculated using the number of non-missing observations for each variable. ACoA–ACA, anterior communicating artery–anterior cerebral artery; PCoA, posterior communicating artery; ACA, anterior cerebral artery; ICA, internal carotid artery; MCA, middle cerebral artery; BA, basilar artery; mWFNS, modified World Federation of Neurosurgical Societies; CRP, C-reactive protein; HDL-C, high-density lipoprotein cholesterol; FHR, fibrinogen-to-HDL-C ratio; MLR, monocyte-to-lymphocyte ratio; nMLR, neutrophil-plus-monocyte-to-lymphocyte ratio; PLR, platelet-to-lymphocyte ratio; NAR, neutrophil-to-albumin ratio; NLR, neutrophil-to-lymphocyte ratio; CAR, C-reactive protein-to-albumin ratio; MHR, monocyte-to-HDL-C ratio; SII, systemic immune-inflammation index; SIRI, systemic inflammation response index; DAR, D-dimer-to-albumin ratio; PNI, prognostic nutritional index; ICU, intensive care unit. Raymond–Roy class refers to angiographic occlusion status after embolization. Antiplatelet or antithrombotic therapy refers to intraoperative tirofiban infusion.

### 3.2 Distribution of functional recovery patterns

**FIGURE 2:**
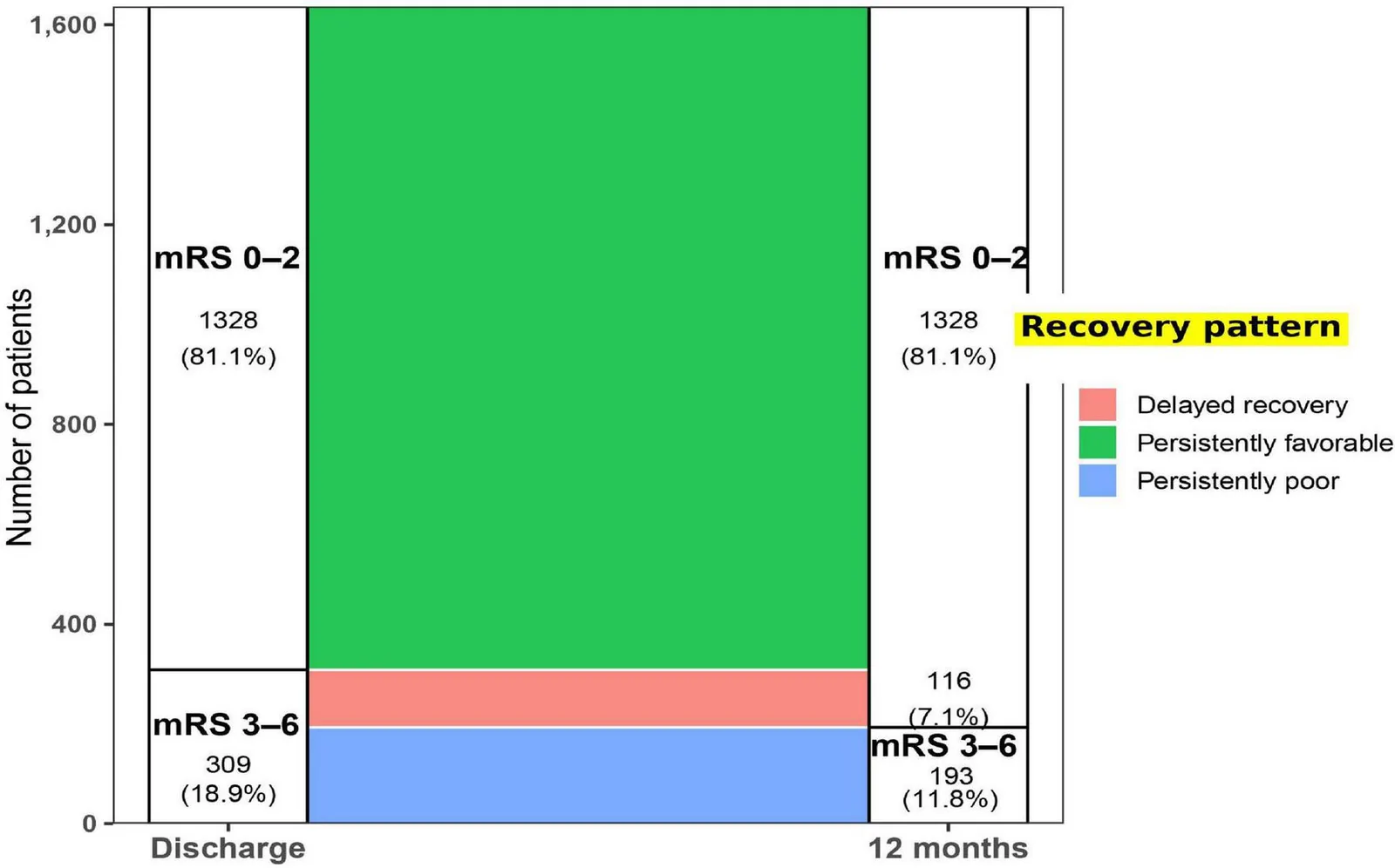
Transition of functional status from discharge to 12 months. Functional status was classified according to the modified Rankin Scale (mRS) as functionally independent (mRS 0–2) or functionally poor (mRS 3–6). The width of each flow represents the number of patients in the corresponding transition pathway. The three recovery patterns are prespecified two-point categories and do not represent modeled longitudinal trajectories. aSAH, aneurysmal subarachnoid hemorrhage.

According to changes in mRS scores from discharge to 12 months, 1,328 patients (81.1%) had mRS scores of 0–2 at both discharge and 12 months and were classified into the persistently favorable group. A total of 116 patients (7.1%) had an mRS score of 3–6 at discharge and 0–2 at 12 months and were classified into the delayed recovery group. The remaining 193 patients (11.8%) had mRS scores of 3–6 at both time points and were classified into the persistently poor group. Among 309 patients with poor function at discharge, 116 (37.5%) had an mRS score of 0–2 at 12 months and 193 (62.5%) had an mRS score of 3–6 (Figure 2).

### 3.3 Clinical characteristics across functional recovery patterns

Baseline characteristics, clinical severity, laboratory parameters, and perioperative variables across the three functional recovery pattern groups are presented in Table 2. Median age was 57.00 years in the persistently favorable group, 61.00 years in the delayed recovery group, and 66.00 years in the persistently poor group (*P* < 0.001). The proportions of mWFNS grades IV–V were 6.9%, 28.5%, and 51.3%, respectively, and the proportions of modified Fisher grade IV were 10.7%, 23.3%, and 42.0%, respectively, (both *P* < 0.001). Hypertension, smoking, alcohol consumption, and several other baseline variables also differed among groups (Table 2).

**TABLE 2:** Baseline characteristics according to recovery patterns.

Variable	Persistently favorable group (*N* = 1328)	Delayed recovery group (*N* = 116)	Persistently poor group (*N* = 193)	*P*-value
Age (years), median (IQR)	57.00 (50.00, 65.00)	61.00 (54.00, 69.00)	66.00 (55.00, 72.00)	**< 0.001**
Sex, *n* (%)				0.817
Male	456 (34.3%)	42 (36.2%)	70 (36.3%)	
Female	872 (65.7%)	74 (63.8%)	123 (63.7%)	
Cerebral hemorrhage (Yes), *n* (%)	41 (3.1%)	4 (3.4%)	7 (3.6%)	0.825
Cerebral ischemia (Yes), *n* (%)	60 (4.5%)	10 (8.6%)	15 (7.8%)	**0.037**
Heart disease (Yes), *n* (%)	49 (3.7%)	10 (8.6%)	16 (8.3%)	**0.002**
Hypertension (Yes), *n* (%)	628 (47.3%)	67 (57.8%)	118 (61.1%)	**< 0.001**
Hyperlipidemia (Yes), *n* (%)	63 (4.7%)	4 (3.4%)	11 (5.7%)	0.665
Diabetes (Yes), *n* (%)	79 (5.9%)	16 (13.8%)	21 (10.9%)	**< 0.001**
Smoking (Yes), *n* (%)	517 (38.9%)	29 (25.0%)	85 (44.0%)	**0.003**
Drinking alcohol (Yes), *n* (%)	467 (35.2%)	25 (21.6%)	83 (43.0%)	**< 0.001**
Aneurysm diameter (mm), median (IQR)	5.00 (3.70, 6.79)	5.10 (3.88, 7.00)	5.15 (3.60, 7.02)	0.614
Neck width (mm), median (IQR)	3.15 (2.47, 4.20)	3.09 (2.21, 4.32)	3.10 (2.40, 4.32)	0.972
Dome-to-neck ratio, median (IQR)	1.40 (1.06, 1.88)	1.36 (1.09, 1.92)	1.47 (1.11, 1.86)	0.492
Parent artery diameter (mm), median (IQR)	2.63 (2.12, 3.23)	2.66 (2.10, 3.37)	2.73 (2.10, 3.37)	0.722
Aneurysm morphology, *n* (%)				0.567
Saccular aneurysm	957 (72.1%)	83 (71.6%)	146 (75.6%)	
Fusiform aneurysm	371 (27.9%)	33 (28.4%)	47 (24.4%)	
Bifurcation status, *n* (%)				**0.029**
Located at an arterial bifurcation	556 (41.9%)	38 (32.8%)	66 (34.2%)	
Not located at an arterial bifurcation	772 (58.1%)	78 (67.2%)	127 (65.8%)	
Aneurysm location, *n* (%)				**< 0.001**
ACoA–ACA	628 (47.3%)	32 (27.6%)	91 (47.2%)	
PCoA	26 (2.0%)	3 (2.6%)	5 (2.6%)	
ACA	39 (2.9%)	8 (6.9%)	7 (3.6%)	
ICA	234 (17.6%)	23 (19.8%)	29 (15.0%)	
MCA	128 (9.6%)	7 (6.0%)	19 (9.8%)	
BA	39 (2.9%)	9 (7.8%)	11 (5.7%)	
Other	234 (17.6%)	34 (29.3%)	31 (16.1%)	
Neutrophils ( × 10^∧^9/L), median (IQR)	7.86 (5.85, 10.59)	9.24 (6.33, 12.26)	9.85 (6.80, 12.40)	**< 0.001**
Lymphocytes ( × 10^∧^9/L), median (IQR)	1.08 (0.74, 1.49)	1.01 (0.70, 1.41)	0.96 (0.65, 1.40)	0.059
Monocytes ( × 10^∧^9/L), median (IQR)	0.59 (0.38, 6.23)	0.78 (0.40, 111.75)	0.62 (0.40, 0.98)	0.152
Platelets ( × 10^∧^9/L), median (IQR)	189.00 (102.00, 248.00)	194.50 (0.99, 251.50)	189.00 (133.00, 259.00)	0.393
C-reactive protein (mg/L), median (IQR)	10.79 (3.52, 23.34)	20.30 (10.22, 32.26)	22.04 (8.06, 44.01)	**< 0.001**
Albumin (g/L), median (IQR)	40.10 (37.10, 42.99)	38.98 (36.38, 42.38)	37.70 (34.50, 41.92)	**< 0.001**
HDL-C (mmol/L), median (IQR)	1.28 (1.15, 1.40)	1.28 (1.16, 1.40)	1.31 (1.17, 1.48)	0.106
Fibrinogen (g/L), median (IQR)	2.98 (2.51, 3.50)	3.18 (2.66, 3.92)	3.18 (2.57, 4.10)	**< 0.001**
D-dimer (mg/L), median (IQR)	1.18 (0.55, 2.50)	1.76 (0.92, 4.40)	2.44 (1.16, 5.32)	**< 0.001**
FHR, median (IQR)	2.34 (1.90, 2.90)	2.44 (2.05, 3.08)	2.47 (1.98, 3.27)	**0.021**
MLR, median (IQR)	0.53 (0.32, 3.76)	0.69 (0.41, 123.89)	0.55 (0.37, 1.25)	0.056
nMLR, median (IQR)	10.15 (5.03, 40.73)	14.85 (6.19, 128.10)	12.58 (6.97, 25.17)	0.053
PLR, median (IQR)	154.64 (63.56, 240.35)	169.63 (1.05, 254.56)	190.28 (112.18, 286.36)	**0.001**
NAR, median (IQR)	0.200 (0.145, 0.268)	0.237 (0.158, 0.330)	0.259 (0.182, 0.332)	**< 0.001**
NLR, median (IQR)	7.33 (4.21, 12.66)	9.78 (5.17, 16.36)	10.02 (5.88, 15.39)	**< 0.001**
CAR, median (IQR)	0.264 (0.085, 0.596)	0.504 (0.205, 0.852)	0.620 (0.205, 1.273)	**< 0.001**
MHR, median (IQR)	0.490 (0.292, 4.748)	0.593 (0.299, 88.716)	0.454 (0.283, 0.807)	0.119
SII, median (IQR)	1017.14 (223.10, 2066.16)	1090.30 (16.14, 2651.79)	1604.69 (753.19, 2859.85)	**< 0.001**
SIRI, median (IQR)	4.65 (2.04, 34.43)	6.93 (2.90, 857.95)	5.54 (3.05, 16.33)	**0.012**
DAR, median (IQR)	0.029 (0.014, 0.065)	0.043 (0.021, 0.119)	0.068 (0.028, 0.137)	**< 0.001**
PNI, median (IQR)	45.83 (42.45, 48.98)	44.70 (41.80, 48.52)	43.00 (38.50, 48.00)	**< 0.001**
mWFNS grade, *n* (%)				**< 0.001**
I	358 (27.0%)	8 (6.9%)	13 (6.7%)	
II	723 (54.4%)	48 (41.4%)	46 (23.8%)	
III	156 (11.7%)	27 (23.3%)	35 (18.1%)	
IV	73 (5.5%)	30 (25.9%)	63 (32.6%)	
V	18 (1.4%)	3 (2.6%)	36 (18.7%)	
Modified Fisher grade, *n* (%)				**< 0.001**
I	204 (15.4%)	6 (5.2%)	8 (4.1%)	
II	791 (59.6%)	52 (44.8%)	46 (23.8%)	
III	191 (14.4%)	31 (26.7%)	58 (30.1%)	
IV	142 (10.7%)	27 (23.3%)	81 (42.0%)	
Intervention approach, *n* (%)				0.549
Coil embolization	618 (46.5%)	57 (49.1%)	99 (51.3%)	
Stent-assisted embolization	701 (52.8%)	58 (50.0%)	94 (48.7%)	
Flow-diverter treatment	9 (0.7%)	1 (0.9%)	0 (0.0%)	
Postoperative complications, *n* (%)				**< 0.001**
Low complication group	1292 (97.3%)	96 (82.8%)	150 (77.7%)	
High complication group	36 (2.7%)	20 (17.2%)	43 (22.3%)	
Perioperative surgical operation, *n* (%)				**< 0.001**
Lumbar puncture	246 (18.5%)	26 (22.4%)	34 (17.6%)	
Lumbar drainage	39 (2.9%)	8 (6.9%)	16 (8.3%)	
External ventricular drainage	17 (1.3%)	8 (6.9%)	36 (18.7%)	
Ventriculoperitoneal shunt	4 (0.3%)	2 (1.7%)	1 (0.5%)	
Craniotomy (decompressive craniectomy)	6 (0.5%)	4 (3.4%)	13 (6.7%)	
No additional neurosurgical procedure	1016 (76.5%)	68 (58.6%)	93 (48.2%)	
ICU length of stay (days), median (IQR)	1.00 (1.00, 2.00)	2.00 (1.00, 3.00)	2.00 (1.00, 4.00)	**< 0.001**
Onset-to-surgery time (days), *n* (%)				0.210
≤ 1	406 (30.6%)	42 (36.2%)	69 (35.8%)	
2–3	318 (23.9%)	24 (20.7%)	48 (24.9%)	
4–7	283 (21.3%)	17 (14.7%)	40 (20.7%)	
> 7	321 (24.2%)	3 3 (28.4%)	36 (18.7%)	
Operative duration (hours), *n* (%)				0.211
≤ 1	410 (30.9%)	43 (37.1%)	61 (31.6%)	
2–3	437 (32.9%)	31 (26.7%)	64 (33.2%)	
4–5	165 (12.4%)	7 (6.0%)	18 (9.3%)	
5–6	45 (3.4%)	3 (2.6%)	8 (4.1%)	
6–7	233 (17.5%)	29 (25.0%)	33 (17.1%)	
> 7	38 (2.9%)	3 (2.6%)	9 (4.7%)	
Treatment status, *n* (%)				0.202
Raymond–Roy I	1163 (87.6%)	93 (80.2%)	165 (85.5%)	
Raymond–Roy II	139 (10.5%)	20 (17.2%)	24 (12.4%)	
Raymond–Roy III	26 (2.0%)	3 (2.6%)	4 (2.1%)	
Antiplatelet or antithrombotic therapy, *n* (%)				**0.015**
Yes	410 (30.9%)	34 (29.3%)	40 (20.7%)	
No	918 (69.1%)	82 (70.7%)	153 (79.3%)	

Data are presented as median [interquartile range (IQR)] for continuous variables and *n* (%) for categorical variables. Percentages were calculated using the number of non-missing observations for each variable. Between-group comparisons were performed using the Kruskal–Wallis test for continuous variables and the chi-square test or Fisher’s exact test for categorical variables, as appropriate. Bold *P-*values indicate *P* < 0.05. Recovery patterns were categorized as persistently favorable, delayed recovery, and persistently poor groups according to the criteria defined in the Methods section. ACoA–ACA, anterior communicating artery–anterior cerebral artery; PCoA, posterior communicating artery; ACA, anterior cerebral artery; ICA, internal carotid artery; MCA, middle cerebral artery; BA, basilar artery; mWFNS, modified World Federation of Neurosurgical Societies; CRP, C-reactive protein; HDL-C, high-density lipoprotein cholesterol; FHR, fibrinogen-to-HDL-C ratio; MLR, monocyte-to-lymphocyte ratio; nMLR, neutrophil-plus-monocyte-to-lymphocyte ratio; PLR, platelet-to-lymphocyte ratio; NAR, neutrophil-to-albumin ratio; NLR, neutrophil-to-lymphocyte ratio; CAR, C-reactive protein-to-albumin ratio; MHR, monocyte-to-HDL-C ratio; SII, systemic immune-inflammation index; SIRI, systemic inflammation response index; DAR, D-dimer-to-albumin ratio; PNI, prognostic nutritional index; ICU, intensive care unit. Raymond–Roy class refers to angiographic occlusion status after embolization. Antiplatelet or antithrombotic therapy refers to intraoperative tirofiban infusion.

Neutrophil count, C-reactive protein, fibrinogen, D-dimer, NLR, CAR, SII, and DAR were higher, whereas albumin and PNI were lower, in the persistently poor group than in the other groups (all *P* < 0.05). No statistically significant differences were observed among the three groups in aneurysm size, neck width, aneurysm morphology, or endovascular treatment modality. Severe postoperative complications, EVD, and ICU length of stay also differed among groups (all *P* < 0.05) (Table 2).

### 3.4 Univariable logistic regression analysis of persistent functional disability

To further identify factors associated with persistent functional disability at 12 months among patients with poor functional status at discharge, univariable logistic regression analysis was performed in the delayed recovery and persistently poor groups. The results showed that age, smoking history, alcohol consumption, aneurysm location, monocyte count, C-reactive protein, albumin, MLR, nMLR, CAR, MHR, SIRI, mWFNS grade, and perioperative neurosurgical procedures were associated with persistent functional disability at 12 months (all *P* < 0.05). No significant associations were observed for the remaining variables, as shown in Table 3.

**TABLE 3:** Univariable logistic regression analysis for persistently poor functional recovery versus delayed recovery.

Variables	β	S.E	Z	*P*	OR (95%CI)
Age (years)	0.02	0.01	2.03	**0.043**	1.02 (1.00 ∼ 1.04)
Sex					
Male					1.00 (Reference)
Female	−0.00	0.24	−0.01	0.991	1.00 (0.62 ∼ 1.61)
Cerebral hemorrhage history	0.05	0.64	0.08	0.935	1.05 (0.30 ∼ 3.68)
Cerebral ischemia history	−0.11	0.43	−0.26	0.791	0.89 (0.39 ∼ 2.06)
Heart disease history	−0.04	0.42	−0.10	0.919	0.96 (0.42 ∼ 2.19)
Hypertension history	0.14	0.24	0.59	0.557	1.15 (0.72 ∼ 1.84)
Hyperlipidemia history	0.53	0.60	0.88	0.377	1.69 (0.53 ∼ 5.44)
Diabetes history	−0.27	0.35	−0.76	0.446	0.76 (0.38 ∼ 1.53)
Smoking history	0.86	0.26	3.32	**< 0.001**	2.36 (1.42 ∼ 3.92)
Drinking alcohol history	1.01	0.27	3.76	**< 0.001**	2.75 (1.62 ∼ 4.65)
Aneurysm diameter (mm)	0.04	0.03	1.17	0.243	1.04 (0.97 ∼ 1.11)
Neck width (mm)	0.01	0.06	0.15	0.881	1.01 (0.90 ∼ 1.13)
Dome-to-neck ratio	0.14	0.15	0.99	0.323	1.15 (0.87 ∼ 1.54)
Parent artery diameter (mm)	0.02	0.12	0.14	0.892	1.02 (0.80 ∼ 1.29)
Aneurysm morphology					
Saccular aneurysm					1.00 (Reference)
Fusiform aneurysm	−0.21	0.27	−0.80	0.426	0.81 (0.48 ∼ 1.36)
Bifurcation status					
Located at an arterial bifurcation					1.00 (Reference)
Not located at an arterial bifurcation	−0.06	0.25	−0.26	0.796	0.94 (0.58 ∼ 1.53)
Aneurysm location					
ACoA–ACA					1.00 (Reference)
PCoA	−0.53	0.76	−0.70	0.481	0.59 (0.13 ∼ 2.59)
ACA	−1.18	0.56	−2.12	**0.034**	0.31 (0.10 ∼ 0.92)
ICA	−0.81	0.35	−2.35	**0.019**	0.44 (0.22 ∼ 0.87)
MCA	−0.05	0.49	−0.10	0.924	0.95 (0.37 ∼ 2.48)
BA	−0.84	0.49	−1.71	0.088	0.43 (0.16 ∼ 1.13)
Other	−1.14	0.32	−3.53	**< 0.001**	0.32 (0.17 ∼ 0.60)
Neutrophils ( × 10^∧^9/L)	0.01	0.03	0.20	0.842	1.01 (0.96 ∼ 1.06)
Lymphocytes ( × 10^∧^9/L)	0.01	0.11	0.05	0.960	1.01 (0.81 ∼ 1.24)
Monocytes ( × 10^∧^9/L)	−0.00	0.00	−2.22	**0.027**	1.00 (0.99 ∼ 1.00)
Platelets ( × 10^∧^9/L)	0.00	0.00	1.15	0.248	1.00 (1.00 ∼ 1.00)
C-reactive protein (mg/L)	0.01	0.00	2.21	**0.027**	1.01 (1.00 ∼ 1.01)
Albumin (g/L)	−0.06	0.02	−2.65	**0.008**	0.94 (0.90 ∼ 0.98)
HDL-C (mmol/L)	0.37	0.39	0.96	0.336	1.45 (0.68 ∼ 3.11)
Fibrinogen (g/L)	0.09	0.10	0.91	0.365	1.10 (0.90 ∼ 1.34)
D-dimer (mg/L)	−0.01	0.01	−0.85	0.393	0.99 (0.97 ∼ 1.01)
FHR	0.04	0.08	0.51	0.612	1.04 (0.89 ∼ 1.22)
MLR	−0.00	0.00	−2.24	**0.025**	1.00 (1.00 ∼ 1.00)
nMLR	−0.00	0.00	−2.17	**0.030**	1.00 (1.00 ∼ 1.00)
PLR	0.00	0.00	1.67	0.095	1.00 (1.00 ∼ 1.00)
NAR	1.22	0.88	1.38	0.169	3.38 (0.60 ∼ 19.13)
NLR	0.00	0.01	0.36	0.717	1.00 (0.98 ∼ 1.03)
CAR	0.35	0.13	2.59	**0.010**	1.41 (1.09 ∼ 1.84)
MHR	−0.00	0.00	−2.34	**0.019**	1.00 (0.99 ∼ 1.00)
SII	0.00	0.00	1.82	0.069	1.00 (1.00 ∼ 1.00)
SIRI	−0.00	0.00	−2.70	**0.007**	1.00 (1.00 ∼ 1.00)
DAR	0.56	0.68	0.83	0.404	1.76 (0.47 ∼ 6.61)
PNI	−0.02	0.02	−1.45	0.146	0.98 (0.95 ∼ 1.01)
mWFNS grade					
I					1.00 (Reference)
II	−0.53	0.49	−1.07	0.286	0.59 (0.22 ∼ 1.55)
III	−0.23	0.52	−0.44	0.662	0.80 (0.29 ∼ 2.20)
IV	0.26	0.50	0.51	0.609	1.29 (0.48 ∼ 3.45)
V	2.00	0.75	2.66	**0.008**	7.38 (1.70 ∼ 32.14)
Modified Fisher grade					
I					1.00 (Reference)
II	−0.41	0.58	−0.71	0.477	0.66 (0.21 ∼ 2.05)
III	0.34	0.58	0.58	0.562	1.40 (0.45 ∼ 4.41)
IV	0.81	0.58	1.39	0.165	2.25 (0.72 ∼ 7.07)
Intervention approach					
Coil embolization					1.00 (Reference)
Stent-assisted embolization	−0.07	0.24	−0.29	0.769	0.93 (0.59 ∼ 1.48)
Flow-diverter treatment	−15.12	882.74	−0.02	0.986	0.00 (0.00 ∼ Inf)
Postoperative complications					
Low complication group					1.00 (Reference)
High complication group	0.32	0.30	1.06	0.288	1.38 (0.76 ∼ 2.48)
Perioperative surgical operation					
Lumbar puncture					1.00 (Reference)
Lumbar drainage	0.42	0.51	0.84	0.400	1.53 (0.57 ∼ 4.12)
External ventricular drainage	1.24	0.47	2.63	**0.009**	3.44 (1.37 ∼ 8.64)
Ventriculoperitoneal shunt	−0.96	1.25	−0.77	0.443	0.38 (0.03 ∼ 4.45)
Craniotomy (decompressive craniectomy)	0.91	0.63	1.45	0.147	2.49 (0.73 ∼ 8.52)
No additional neurosurgical procedure	0.04	0.31	0.15	0.883	1.05 (0.57 ∼ 1.90)
ICU length of stay (days)	0.05	0.03	1.67	0.095	1.05 (0.99 ∼ 1.11)
Onset-to-surgery time (days)					
≤ 1					1.00 (Reference)
2–3	0.20	0.32	0.62	0.536	1.22 (0.65 ∼ 2.27)
4–7	0.36	0.35	1.03	0.304	1.43 (0.72 ∼ 2.84)
> 7	−0.41	0.31	−1.32	0.187	0.66 (0.36 ∼ 1.22)
Operative duration (hours)					
≤ 1					1.00 (Reference)
2–3	0.38	0.30	1.27	0.205	1.46 (0.81 ∼ 2.60)
4–5	0.59	0.49	1.22	0.223	1.81 (0.70 ∼ 4.72)
5–6	0.63	0.71	0.89	0.371	1.88 (0.47 ∼ 7.50)
6–7	−0.22	0.32	−0.68	0.495	0.80 (0.43 ∼ 1.51)
> 7	0.75	0.70	1.08	0.282	2.11 (0.54 ∼ 8.27)
Treatment status					
Raymond–Roy I					1.00 (Reference)
Raymond–Roy II	−0.39	0.33	−1.19	0.235	0.68 (0.35 ∼ 1.29)
Raymond–Roy III	−0.29	0.77	−0.37	0.712	0.75 (0.16 ∼ 3.43)
Antiplatelet or antithrombotic therapy					
Yes					1.00 (Reference)
No	0.46	0.27	1.71	0.088	1.59 (0.93 ∼ 2.69)

Univariable logistic regression was used to evaluate factors associated with persistently poor functional recovery compared with delayed recovery after endovascular treatment for aneurysmal subarachnoid hemorrhage. The delayed recovery group was used as the reference outcome group, and the persistently poor group was defined as the event group. For cerebral hemorrhage, cerebral ischemia, heart disease, hypertension, hyperlipidemia, diabetes, smoking, and drinking alcohol, the reference category was the absence of the corresponding medical history or exposure. For continuous variables, ORs were calculated per 1-unit increase. Bold values indicate statistical significance (*P* < 0.05). ACoA–ACA, anterior communicating artery–anterior cerebral artery; PCoA, posterior communicating artery; ACA, anterior cerebral artery; ICA, internal carotid artery; MCA, middle cerebral artery; BA, basilar artery; mWFNS, modified World Federation of Neurosurgical Societies; HDL-C, high-density lipoprotein cholesterol; FHR, fibrinogen-to-HDL-C ratio; MLR, monocyte-to-lymphocyte ratio; nMLR, neutrophil-plus-monocyte-to-lymphocyte ratio; PLR, platelet-to-lymphocyte ratio; NAR, neutrophil-to-albumin ratio; NLR, neutrophil-to-lymphocyte ratio; CAR, C-reactive protein-to-albumin ratio; MHR, monocyte-to-HDL-C ratio; SII, systemic immune-inflammation index; SIRI, systemic inflammation response index; DAR, D-dimer-to-albumin ratio; PNI, prognostic nutritional index; ICU, intensive care unit.

### 3.5 Correlation and multicollinearity assessment of candidate variables

**FIGURE 3:**
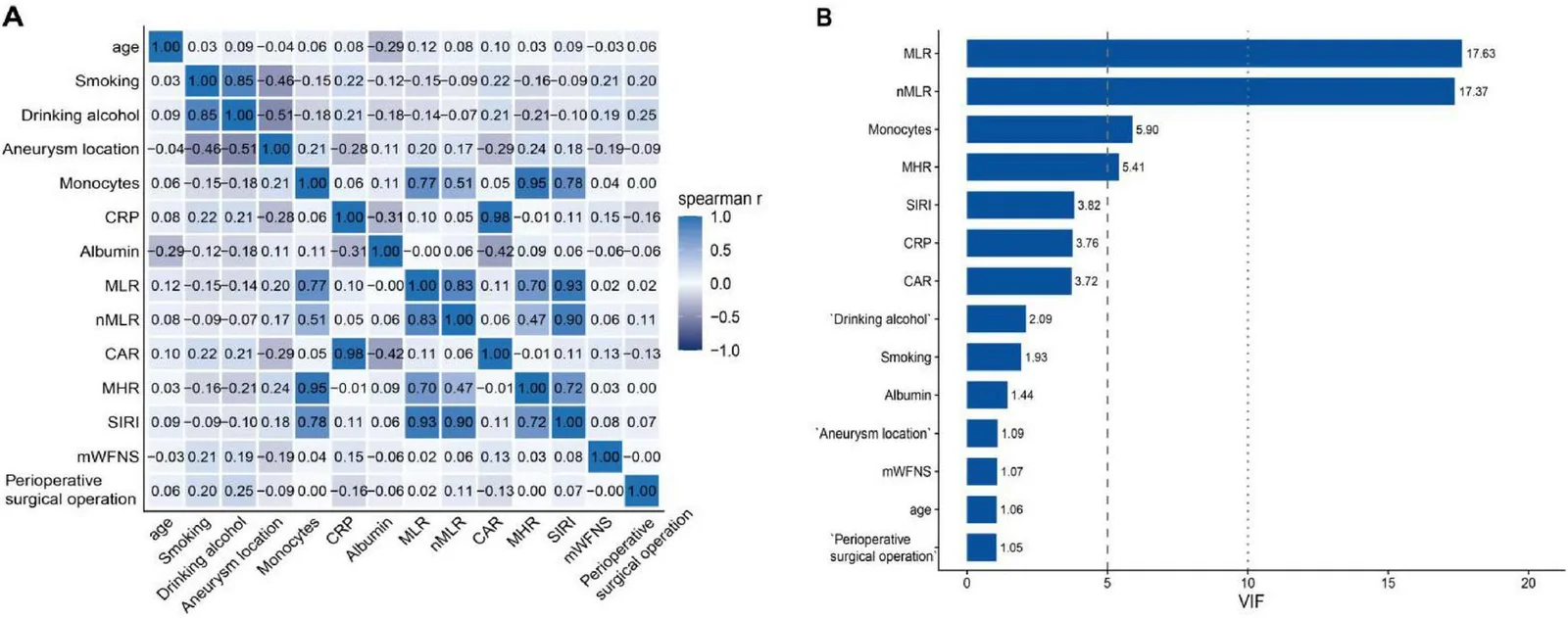
Correlation and multicollinearity assessment of candidate variables. **(A)** Spearman correlation matrix of variables with *P* < 0.05 in the univariable logistic regression analysis. Color intensity indicates the magnitude and direction of the correlation coefficient. **(B)** Variance inflation factor (VIF) analysis of candidate variables. The dashed line indicates the VIF threshold of 10; VIF > 10 was considered indicative of severe multicollinearity. CRP, C-reactive protein; CAR, C-reactive protein-to-albumin ratio; MHR, monocyte-to-HDL-C ratio; MLR, monocyte-to-lymphocyte ratio; nMLR, neutrophil-plus-monocyte-to-lymphocyte ratio; SIRI, systemic inflammation response index; VIF, variance inflation factor.

Candidate variables with *P* values < 0.05 in the univariable analysis were evaluated using Spearman correlation analysis and variance inflation factors (VIFs). Correlations were observed between smoking history and alcohol consumption, CRP and CAR, monocyte count and MHR, and nMLR and SIRI (Figure 3A). The VIF values for MLR and nMLR were 17.63 and 17.37, respectively, and exceeded the prespecified threshold of 10; these variables were therefore excluded from the multivariable models. The remaining candidate variables had VIF values < 10 and were included in the multivariable analysis (Figure 3B).

### 3.6 Multivariable logistic regression analysis of factors associated with persistent functional disability

Variables with *P-*values < 0.05 in the univariable analysis and without evidence of severe multicollinearity were entered into the multivariable logistic regression models. The crude model included all eligible candidate variables. Model 2 was additionally adjusted for sex, whereas the fully adjusted model further accounted for a history of cerebral hemorrhage, cerebral ischemia, heart disease, hypertension, hyperlipidemia, and diabetes mellitus. The results are presented in Table 4.

**TABLE 4:** Multivariable logistic regression for persistent disability versus delayed recovery.

Variable	Crude OR (95% CI)	Crude *P*-value	Adjusted OR (95% CI)	Adjusted *P*-value	Fully adjusted OR (95% CI)	Fully adjusted *P*-value
Age	1.03 (1.00, 1.05)	0.040	1.02 (1.00, 1.05)	0.085	1.02 (0.99, 1.05)	0.141
Smoking	0.92 (0.33, 2.57)	0.875	1.11 (0.38, 3.16)	0.838	1.30 (0.43, 3.84)	0.635
Drinking alcohol	2.19 (0.72, 6.80)	0.166	2.10 (0.70, 6.47)	0.186	1.88 (0.61, 5.92)	0.272
Aneurysm location						
ACoA–ACA (Reference)	Reference		Reference		Reference	
PCoA vs. ACoA–ACA	0.91 (0.14, 7.89)	0.924	0.91 (0.14, 8.07)	0.928	0.85 (0.13, 7.82)	0.876
ACA vs. ACoA–ACA	0.57 (0.16, 1.94)	0.363	0.56 (0.16, 1.91)	0.351	0.54 (0.15, 1.88)	0.328
ICA vs. ACoA–ACA	1.13 (0.47, 2.76)	0.783	1.13 (0.47, 2.76)	0.780	1.20 (0.49, 2.98)	0.688
MCA vs. ACoA–ACA	2.23 (0.78, 7.00)	0.147	2.24 (0.78, 7.01)	0.146	2.29 (0.79, 7.24)	0.140
BA vs. ACoA–ACA	0.74 (0.24, 2.30)	0.592	0.74 (0.24, 2.32)	0.599	0.71 (0.22, 2.25)	0.553
Other vs. ACoA–ACA	0.62 (0.28, 1.37)	0.237	0.61 (0.28, 1.36)	0.227	0.65 (0.29, 1.45)	0.286
Monocytes	1.02 (1.00, 1.04)	0.047	1.02 (1.00, 1.04)	0.038	1.02 (1.00, 1.04)	0.043
CRP	0.99 (0.95, 1.02)	0.387	0.98 (0.95, 1.01)	0.348	0.98 (0.95, 1.01)	0.342
Albumin	0.99 (0.93, 1.02)	0.639	0.98 (0.92, 1.02)	0.634	0.98 (0.92, 1.02)	0.574
CAR	1.69 (0.65, 6.74)	0.362	1.81 (0.68, 7.70)	0.327	1.79 (0.68, 7.71)	0.336
MHR	0.98 (0.96, 1.00)	0.086	0.98 (0.96, 1.00)	0.081	0.98 (0.96, 1.00)	0.097
SIRI, per IQR increase	0.99 (0.98, 1.00)	0.028	0.99 (0.98, 1.00)	0.023	0.99 (0.98, 1.00)	0.019
mWFNS	1.57 (1.23, 2.02)	< 0.001	1.59 (1.25, 2.06)	< 0.001	1.67 (1.29, 2.18)	< 0.001
Perioperative surgical operation						
No additional neurosurgical procedure	Reference		Reference		Reference	
Lumbar puncture	1.19 (0.60, 2.37)	0.619	1.29 (0.65, 2.61)	0.471	1.26 (0.62, 2.58)	0.531
Lumbar drainage	1.53 (0.56, 4.40)	0.416	1.48 (0.54, 4.26)	0.452	1.52 (0.55, 4.44)	0.427
External ventricular drainage	3.63 (1.47, 10.05)	0.008	3.78 (1.52, 10.56)	0.007	3.71 (1.47, 10.49)	0.008
Ventriculoperitoneal shunt	0.56 (0.02, 6.66)	0.653	0.54 (0.02, 6.39)	0.630	0.67 (0.03, 8.21)	0.757
Craniotomy (decompressive craniectomy)	2.88 (0.84, 12.20)	0.114	2.87 (0.84, 12.07)	0.114	2.60 (0.73, 11.33)	0.163

Multivariable logistic regression was performed to identify independent factors associated with persistent disability compared with delayed recovery. Outcome: persistent disability versus delayed recovery. The event category was defined as persistent disability. Therefore, OR > 1 indicates higher odds of persistent disability. Variables with *P* < 0.05 in the univariable analysis were entered into the multivariable model. Model 1 included these significant variables without additional covariate adjustment. Adjusted model was further adjusted for Sex. Fully adjusted model was further adjusted for medical history, including Cerebral hemorrhage history, Cerebral ischemia history, Heart disease history, Hypertension history, Hyperlipidemia history, and Diabetes history. SIRI, systemic immune-inflammation response index; MHR, monocyte-to-high-density lipoprotein cholesterol ratio; CAR, C-reactive protein-to-albumin ratio; mWFNS, modified World Federation of Neurosurgical Societies grade. Smoking and Drinking alcohol were analyzed as yes versus no. Categorical variables are presented relative to their reference categories. *P-*values were two-sided.

**FIGURE 4:**
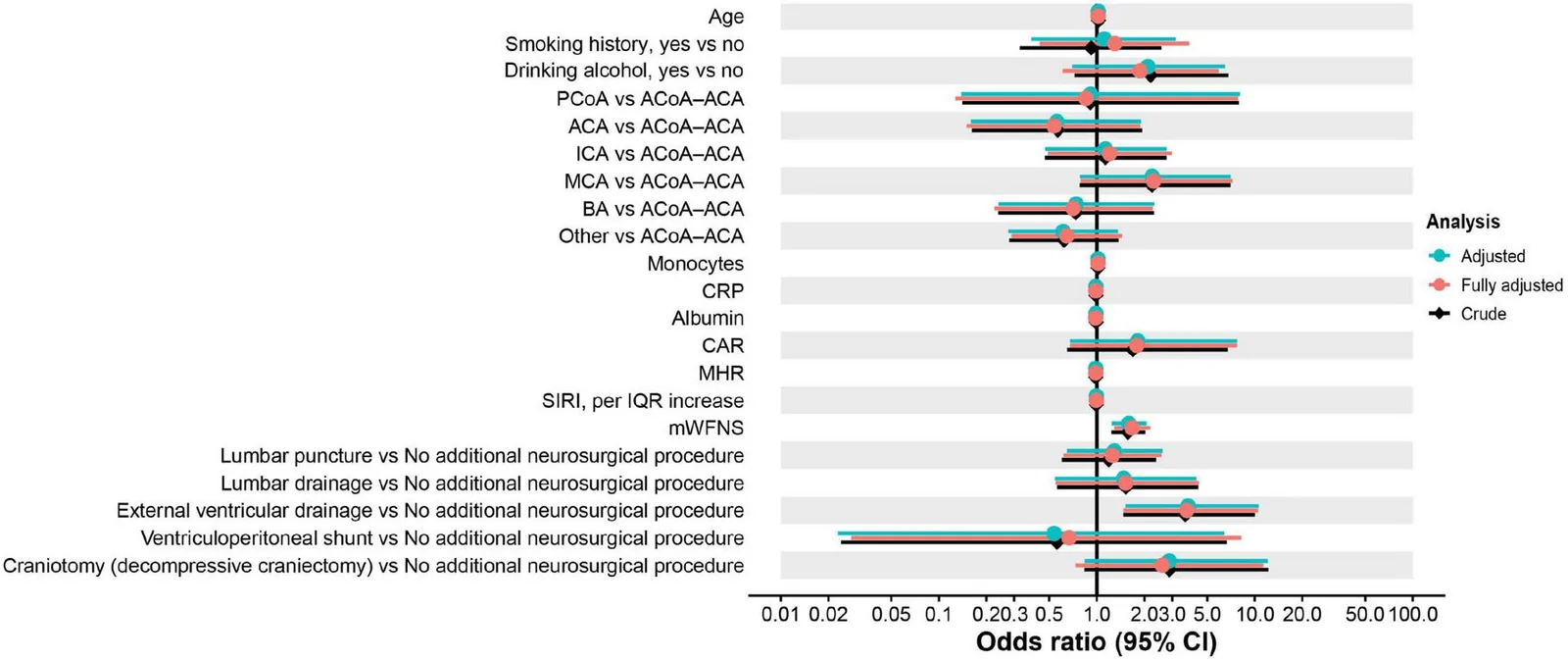
Forest plot of multivariable logistic regression for factors associated with persistent disability. Crude included candidate variables with *P* < 0.05 in the univariable analysis and without severe multicollinearity. Adjusted was further adjusted for sex. The fully adjusted model was additionally adjusted for medical history, including history of cerebral hemorrhage, cerebral ischemia, heart disease, hypertension, hyperlipidemia, and diabetes. The delayed recovery group was used as the reference outcome group, and the persistently poor group was defined as the event group. An OR > 1 indicates higher odds of persistent disability. Dots represent ORs, and horizontal lines represent 95% confidence intervals. mWFNS, modified World Federation of Neurosurgical Societies; SIRI, systemic inflammation response index; CRP, C-reactive protein; CAR, C-reactive protein-to-albumin ratio; MHR, monocyte-to-HDL-C ratio; OR, odds ratio; CI, confidence interval.

In the fully adjusted model, a higher monocyte count (OR = 1.02, 95% CI: 1.00–1.04; *P* = 0.043), a higher mWFNS grade (OR = 1.67, 95% CI: 1.29–2.18; *P* < 0.001), and EVD (OR = 3.71, 95% CI: 1.47–10.49; *P* = 0.008) were associated with increased odds of persistent functional disability at 12 months. Each interquartile-range increase in SIRI was associated with lower odds of persistent functional disability (OR = 0.99, 95% CI: 0.98–1.00; *P* = 0.019). Age, smoking history, alcohol consumption, aneurysm location, CRP, albumin, CAR, and MHR did not remain associated with persistent functional disability after full adjustment (Figure 4 and Table 4).

## 4 Discussion

Previous studies of prognosis after aneurysmal subarachnoid hemorrhage (aSAH) have predominantly focused on predicting unfavorable outcomes at predefined time points. However, functional outcome after aSAH is not static. In a longitudinal study, Hammer et al. demonstrated that modified Rankin Scale (mRS) scores continued to improve after hospital discharge, with varying degrees of functional recovery occurring both from discharge to 6 months and from 6 months to 1 year (Hammer et al., 2020). Similarly, the long-term follow-up study by Lee et al. suggested that functional status among aSAH survivors may continue to improve for several years after the initial event (Lee et al., 2025). Therefore, defining prognosis solely on the basis of functional status at discharge or at a single follow-up time point may underestimate the recovery potential of some patients and may fail to distinguish those with delayed recovery from those with persistently poor outcomes.

Building on previous studies based on static outcome assessments, the present study characterized functional recovery patterns according to changes in mRS scores between discharge and 12 months, thereby providing a trajectory-based perspective on long-term outcomes after endovascular treatment for aSAH. We observed substantial heterogeneity in post-discharge functional recovery. Notably, among patients with poor functional status at discharge, 37.5% regained functional independence by 12 months, whereas 62.5% remained functionally disabled. These findings are consistent with previous evidence indicating that functional recovery may continue after aSAH. Navi et al. reported that a substantial proportion of patients with disability at discharge after subarachnoid hemorrhage regained functional independence within 6 months (Navi et al., 2012), while Lindner et al. observed continued improvement in mRS scores up to 1 year after discharge among patients with recovery potential (Lindner et al., 2023). On this basis, identifying clinical factors associated with different recovery patterns may help distinguish patients with the potential for delayed recovery from those at high risk of persistent functional disability.

A higher mWFNS grade was independently associated with persistent functional disability and was one of the most clinically interpretable findings of this study. Previous studies have consistently shown that WFNS or Hunt–Hess grade is strongly associated with short- and long-term outcomes after aSAH (Rosengart et al., 2007), and a systematic review identified early clinical grade as an important determinant of functional outcome in high-grade aSAH (de Winkel et al., 2022). Our findings extend this evidence by showing that higher admission mWFNS grade remained associated with persistent disability even among patients who already had poor functional status at discharge. This association likely reflects the greater burden of early brain injury and its lasting effects on neurological recovery. Therefore, a high mWFNS grade may help identify patients with limited post-discharge recovery potential despite successful aneurysm occlusion and clinical stabilization.

EVD was associated with persistent disability at 12 months (Chung et al., 2018); however, this finding should not be interpreted as evidence that EVD is an independent biological determinant of poor recovery or that the procedure itself causes harm. EVD is generally required in patients with acute hydrocephalus, intraventricular hemorrhage, or intracranial hypertension and may therefore function as a marker of greater initial disease severity, ventricular involvement, and a more complicated neurocritical-care course. Accordingly, the adjusted association is more plausibly interpreted as residual information about disease severity and treatment indication than as a direct effect of EVD (Nelson et al., 2022).

The findings for inflammatory indices should be interpreted cautiously. The association of monocyte count with persistent disability was small, whereas the direction of the SIRI association differed from previous reports and the estimate was close to the null. These results should be regarded as exploratory statistical associations rather than evidence for individual-level clinical prediction or a specific biological mechanism. Repeated measurements and external validation are needed(Geraghty et al., 2021; Hou et al., 2023).

From a clinical perspective, the principal contribution of this study lies not in identifying a single prognostic factor, but in proposing a risk-stratification framework that more closely reflects the dynamic recovery process after aSAH. Whereas previous studies have primarily focused on predicting unfavorable functional outcomes at predefined time points, the present study further examined the likelihood of subsequent recovery among patients with poor functional status at discharge. This question is particularly relevant to clinical decision-making.

For patients who are functionally impaired at discharge but have a relatively low mWFNS grade, have not undergone EVD, and exhibit a lower systemic inflammatory burden, clinicians should avoid prematurely pessimistic prognostic counseling and should actively facilitate structured rehabilitation and follow-up. By contrast, patients with a high mWFNS grade, EVD requirement, substantial inflammatory burden, or poor nutritional status may warrant more intensive long-term assessment of physical function, cognition, gait, and quality of life. Such a recovery pattern–based management strategy may extend prognostic assessment beyond the prediction of outcomes at isolated time points toward longitudinal care guided by individual recovery trajectories.

This study has several limitations. First, its retrospective design may have introduced selection bias, residual confounding, and confounding by indication. Second, functional recovery was assessed using the mRS at only discharge and 12 months because intermediate assessments were incomplete across centers. This two-time-point approach may not capture nonlinear changes in recovery, and the mRS itself does not fully reflect cognitive, emotional, or social outcomes. Third, detailed information on hydrocephalus severity, EVD management, discharge destination, and post-discharge rehabilitation was not consistently available, which may have resulted in unmeasured confounding. Fourth, inflammatory markers were primarily measured at a single acute-phase time point; therefore, the associations of monocyte count and SIRI should be considered exploratory and require further validation. Fifth, the regression analyses were intended to estimate associations rather than develop an individual-level prediction model. Finally, because all patients were treated at tertiary centers in China and the cohort was restricted to patients undergoing endovascular treatment, whether these findings can be generalized to other healthcare settings and to patients treated with microsurgical clipping remains uncertain and requires further validation.

In this multicenter cohort of endovascularly treated patients with aSAH, paired discharge and 12-month mRS assessments demonstrated substantial heterogeneity in post-discharge recovery. Higher mWFNS grade and EVD were associated with persistent disability, whereas the associations of inflammatory markers remained exploratory. These findings underscore the importance of considering recovery potential when counseling and planning follow-up and rehabilitation for patients with initially poor functional status.

## Data Availability

The original contributions presented in this study are included in this article/supplementary material, further inquiries can be directed to the corresponding authors.
